# Disparities in bone density measurement history and osteoporosis medication utilisation in Swiss women: results from the Swiss Health Survey 2007

**DOI:** 10.1186/1471-2474-14-10

**Published:** 2013-01-05

**Authors:** Rita Born, Marcel Zwahlen

**Affiliations:** 1 Institute of Social and Preventive Medicine, Bern University, Bern, Switzerland

**Keywords:** Bone density measurement, Osteoporosis, Medication, Sociodemographic, Socioeconomic, Health survey

## Abstract

**Background:**

Although factors associated with the utilisation of bone density measurement (BDM) and osteoporosis treatment have been regularly assessed in the US and Canada, they have not been effectively analysed in European countries. This study assessed factors associated with the utilisation of BDM and osteoporosis medication (OM) in Switzerland.

**Methods:**

The Swiss Health Survey 2007 data included self-reported information on BDM and OM for women aged 40 years and older who were living in private households. Multivariable logistic regression analysis was used to identify sociodemographic, socioeconomic, healthcare-related and osteoporosis risk factors associated with BDM and OM utilisation.

**Results:**

The lifetime prevalence of BDM was 25.6% (95% CI: 24.3-26.9%) for women aged 40 years and older. BDM utilisation was associated with most sociodemographic factors, all the socioeconomic and healthcare-related factors, and with major osteoporosis risk factors analysed. The prevalence of current OM was 7.8% (95% CI: 7.0-8.6%) and it was associated with some sociodemographic and most healthcare-related factors but only with one socioeconomic factor.

**Conclusions:**

In Swiss women, ever having had a BDM and current OM were low and utilisation disparities exist according to sociodemographic, socioeconomic and healthcare-related factors. This might foster further health inequalities. The reasons for these findings should be addressed in further studies of the elderly women, including those living in institutions.

## Background

In Switzerland and especially in women, the prevalence of osteoporosis is high and expected to increase because of the aging of the population [1]. In 2000, 316,000-348,000 women and men were estimated to have osteoporosis, with this prevalence predicted to increase by 25% by 2020 (to 395,000-437,000 people) [2]. Osteoporosis-related fragility fractures cause significant individual, societal and economic burdens [2,3].

Fracture risk assessment, bone mineral density measurement (BDM), and subsequent interventions (including medical treatment) can reduce the risk of osteoporosis and osteoporotic fractures [4-6].

General screening is not officially recommended in Switzerland, although a “screen & treat” strategy against osteoporosis was assessed to be cost-effective for women aged 70 years and older [7,8]. In 2003, the Swiss Union Against Osteoporosis (SVGO) recommended BDM for diagnostic purposes after a fragility fracture, and it endorsed screening prior to fractures when at least one major or two minor risk factors were present [9]. In Switzerland, compulsory health insurance covers BDM for diagnostic purposes but not for screening purposes, except for individuals with at least one major risk factor, such as long-term glucocorticoid therapy, a gastrointestinal disease, hypogonadism, hyperparathyroidism, or osteogenesis imperfecta [10].

In contrast to the SVGO, the Canadian Scientific Advisory Council of Osteoporosis recommended routine bone density screening for all individuals at age 65, and screening prior to age 65 for those with certain risk factors. In addition, BDM was recommended for diagnostic purposes after a fragility fracture [11]. Similarly, the US Preventive Services Task Force advised women aged 65 years and older or aged 60 years and older with an increased osteoporosis risk to be routinely screened for osteoporosis using dual-energy x-ray absorptiometry (DXA) [12,13]. Despite the existence of these recommendations, surveys among women in Canada and the US showed that having had a BDM was associated with sociodemographic factors and socioeconomic status, and it was only weakly associated with osteoporosis risk factors [14-16]. Similar associations were observed for taking osteoporosis medication (OM), which was recommended for postmenopausal women with low bone density in Canada and the US [17,18].

Little is known about factors associated with BDM history and OM utilisation in Switzerland or other European countries. However, the findings in Canada and the US led us to hypothesise that similar disparities may exist in Swiss women, even though BDM and OM are, with some exceptions, reimbursed [10,19]. This hypothesis is also supported by results of the Swiss Health Survey in 2002 showing, for example, variations in the utilisation of general healthcare services and cancer screening modalities according to sociodemographic and socioeconomic characteristics of the Swiss resident population [20].

The aim of our study was to describe the Swiss situation with regard to history of BDM and current OM utilisation in Swiss women based on the most recent Swiss Health Survey (SHS) data. Specifically, we sought to assess whether BDM and OM utilisation in women vary with respect to sociodemographic and socioeconomic factors, healthcare utilisation, and/or established risk factors for osteoporosis and osteoporotic fractures that justify a BDM according to SVGO recommendations.

## Methods

### Study design and data collection

The methods of the SHS 2007, including sampling, weighting and adjustment for non-response are described in detail elsewhere [21]. Briefly, the SHS 2007 was conducted by the Swiss Federal Statistical Office (SFSO). The survey data are representative for Swiss residents aged 15 years and older who lived in private households with a registered landline phone in 2007. Institutionalised adults (e.g., those in nursing homes, hospitals, prisons, and monasteries), adults without a landline phone, individuals who resided in Switzerland for 3 months or less and those who did not speak German, French or Italian were, by design, excluded [21,22].

A random sample was obtained by stratified selection in two stages; in the first stage, households were randomly selected in each canton with oversampling in 14 cantons, whereas in the second stage, one person was randomly selected in each household. The sampling list consisted of 30,179 addresses of households, of which 1,847 addresses were not valid. Of the remaining 28,332 households, 6,158 households were unavailable, and 3,414 persons refused to participate, resulting in a sample of 18,760 interviewed persons (response rate 66%).

The SHS participants were interviewed via a computer-assisted phone interview (CATI) conducted by the Institute MIS-Trend SA in Lausanne and Gümligen on behalf of the SFSO. The SFSO constructed a weighting for each record in the SHS to account for the oversampling in certain cantons and for non-responses of households and individuals. The marginal distributions of the SHS respondents weighted with respect to sex, age group, nationality, language region and canton of residence were calibrated to the distributions of the 2006 Swiss resident population, excluding those living in institutions.

### Questionnaire

The CATI questionnaire included close to 400 questions grouped in 69 domains, covering areas such as sociodemographic and socioeconomic status, health status, health-related behaviours and attitudes, and health services utilisation [23].

#### Outcome variables

In contrast to the prior SHSs in 1992, 1997 and 2002, the SHS 2007 contained questions pertinent to the history of BDM and OM utilisation.

All women aged 40 years and older were asked whether they had ever had a BDM (yes or no). In addition, all women older than 40 years (and not only those having had a BDM) were asked how often they had taken medications against osteoporosis in the last 7 days (daily, several times, about once or never) and in case of treatment, if the medication had been prescribed by a physician (yes or no). Whether a woman currently takes hormone replacement therapy (HRT) was a separate question in the SHS. Taking HRT was not classified as an OM. With this information, the variable of physician prescribed OM in the last 7 days (yes or no) was created.

#### Sociodemographic and socioeconomic factors

The sociodemographic variables assessed were age (grouped into 40–49 years, 50–59 years, 60–69 years, 70–79 years, and ≥80 years), nationality (Swiss, Swiss citizen with dual nationality, or other), language region (German, French or Italian), region (Lake Geneva, Midland, Northwest, Zurich, East, Central or Ticino) and residential area (urban or rural). The socioeconomic variables evaluated were type of hospitalisation insurance (public, semiprivate or private), education level (compulsory, secondary or tertiary) and equivalent per capita income grouped in tertiles (lower, middle or upper).

The variables for region, residential area, education level and equivalent per capita income were indexed, calculated and supplied by the SFSO, and they are described elsewhere [24].

#### Health and healthcare-related factors

The healthcare-related factors assessed were number of physician visits in the past 12 months (no visits, 1 visit, 2–6 visits, or >6 visits) and a gynaecologist check-up in the last 12 months (yes or no). Self-assessed current health status was obtained from the question “How is your health in general?” with five response options from “very bad” to “very good”. The question on self-assessed health status is part of the “Minimum European Health Module” (MEHM). The category “very good” was combined with “good”, and “very bad” with “bad”. Whether the individual had a health-oriented lifestyle (yes or no) was derived from the question “How important is health for you? You have here 3 different opinions. Please state which one is closest to your own opinion: 1. I live without taking care of possible consequences for my health. 2. Ideas on the maintenance of my health influence my lifestyle. 3. Health-related considerations determine mainly how I live.”

The respondents were coded to have a health-oriented lifestyle if they gave answer 2 or 3. In addition, information on having ever had a test for cancer in the absence of symptoms was used to create a variable on cancer screening ever performed (yes or no) with regard to mammography and colon cancer screening. Furthermore, a variable describing the type of OM (none, self-ordered, prescribed, or HRT) was constructed using information on OM and HRT.

#### Osteoporosis risk factors

The risk factors assessed were smoking (no, former or not daily, or yes), daily tobacco consumption (none, 0–9 cigarettes, or ≥10 cigarettes), daily alcohol intake (none, 0–30 g, or >30 g) [6], underweight defined as body mass index below 20 kg/m^2^ (yes or no) [6] and a history of falls in the past 12 months (yes or no). The question on falls was restricted to individuals aged 60 years and older. Risk factors and their levels were defined taking the FRAX calculation tool into account [6].

Level of physical activity (inactive, partially active, or active) was included as protective factor. These categories of physical activity were defined by the SFSO as described elsewhere [24].

### Statistical analysis

All analyses were weighted using the record weightings as provided by the SFSO [21]. Survey commands (svy prefix) of the statistical software Stata version 11.2 (Stata Corp, College Station, Texas, US.) were used to perform the weighted analyses.

For each variable of interest, the number of respondents in each group, the (weighted) percentages of the population in each group and the (weighted) percentages of the population with the outcome of interest per group were calculated. The significance of intergroup differences was tested using the Pearson γ^2^-test (significance level p<0.05).

The crude associations of variables of interest with history of BDM or current OM were first evaluated using univariable logistic regression analyses. To adjust for confounders and to identify variables independently associated with the outcome of interest, multivariable logistic regression analyses were performed. Based on the findings in the US and in Canada, three distinct regression models were fitted; the first two models included either sociodemographic covariates (age, region, residential area) or socioeconomic covariates (education level, per capita income, type of hospitalisation insurance), and the third model adjusted for both groups (age, region, residential area, education level, per capita income, hospitalisation insurance type). The significance of associations was examined using the Wald test (significance level p<0.05).

The reference groups were the lowest level for the ordered variables, the group with the highest number of observations for the not-ordered categorical variables and the level without the corresponding characteristic of interest for the dichotomous variables. For age, the reference group was those aged 50–59 years. Ethical approval was not required for this analysis of data, which are available to research institutions according to the ordinance on federal statistical monitoring activities and surveys [25].

## Results

### Bone density measurement utilisation in women

Of the 7,052 female respondents aged 40 years and older, 327 were excluded due to missing information on BDM utilisation, leaving 6,725 women for analysis (95.4%).

#### Lifetime prevalence of BDM

In 2007, 25.6% (95% CI: 24.3-26.9%) of female residents aged 40 years and older had performed a BDM at least once (Table 1). BDM prevalence was highest in the 60–79 years age groups (41.8%) and lowest in the 40–49 years age group (5.8%). It was highest in Ticino (32.0%) and lowest in the Central region (17.8%), and it was higher in urban areas (26.7%). Additionally, utilisation rate was highest in the Italian-speaking region (31.4%), which corresponds approximately to the Ticino, and lowest in the German-speaking region (23.8%). Furthermore, ever having had a BDM was higher for women with a higher income (29.8%), for women with private hospitalisation insurance (36.6%) and for those with more doctor visits in the past year. In addition it was higher (33.7%) in those who had ever undergone a breast or colon cancer screen.

**Table 1:** BDM in women according to sociodemographic, socioeconomic, risk and healthcare-related factors

**BDM**	**Sample**^**a**^	**Population**		** Proportion with BDM**		** Unadjusted model**		** Adjusted model**^**b**^	
**Ever performed**	**N**	**%**	**(95% CI)**	**%**	**(95% CI)**	**crude OR**	**(95% CI)**	**adjusted OR**	**(95% CI)**
**Total**	6,725	100.0%		25.6%	(24.3-26.9%)				
**Sociodemographic factors**									
**Age group**				***p=0.0000***		***p=0.0000***		***p=0.0000***	
40-49 years	1,760	31.1%	(29.7-32.6%)	5.8%	(4.6-7.3%)	0.19	(0.14-0.25)	0.21	(0.16-0.29)
50-59 years	1,536	24.9%	(23.6-26.3%)	24.8%	(22.2-27.5%)	1.00	(ref.)	1.00	(ref.)
60-69 years	1,629	21.5%	(20.4-22.8%)	41.8%	(38.8-44.8%)	2.18	(1.80-2.63)	2.48	(2.02-3.03)
70-79 years	1,195	14.8%	(13.9-15.9%)	41.8%	(38.4-45.4%)	2.18	(1.78-2.67)	2.70	(2.16-3.38)
≥80 years	605	7.6%	(6.9-8.3%)	32.0%	(27.5-36.8%)	1.43	(1.10-1.85)	1.78	(1.34-2.37)
mean age (years)		58.5	(58.2-58.9)						
**Nationality**				***p=0.1109***		***p=0.0916***		***p=0.0074***	
Swiss citizen	5,502	78.3%	(76.9-79.6%)	25.5%	(24.2-27.0%)	1.00	(ref.)	1.00	(ref.)
Swiss dual citizen	712	10.1%	(9.3-11.0%)	29.2%	(25.4-33.4%)	1.20	(0.98-1.48)	1.43	(1.12-1.83)
others	510	11.6%	(10.5-12.9%)	22.6%	(18.3-27.5%)	0.85	(0.65-1.12)	1.32	(0.96-1.83)
unknown	1								
**Language region**				***p=0.0000***		***p=0.0000***		***p=0.0000***	
German	4,148	72.8%	(72.0-73.5%)	23.8%	(22.3-25.4%)	1.00	(ref.)	1.00	(ref.)
French	1,965	22.1%	(21.5-22.8%)	30.0%	(27.6-32.6%)	1.37	(1.18-1.59)	1.58	(1.34-1.87)
Italian	612	5.1%	(4.8-5.4%)	31.4%	(27.3-35.8%)	1.46	(1.18-1.81)	1.56	(1.20-2.03)
**Region**				***p=0.0000***		***p=0.0000***		***p=0.0000***	
Lake Geneva	1,190	17.2%	(16.6-17.8%)	30.0%	(27.0-33.1%)	1.29	(1.06-1.58)	1.37	(1.09-1.72)
Midland	1,750	23.0%	(22.2-23.7%)	24.8%	(22.4-27.5%)	1.00	(ref.)	1.00	(ref.)
Northwest	752	14.9%	(14.3-15.5%)	28.8%	(25.2-32.6%)	1.22	(0.97-1.53)	1.13	(0.86-1.46)
Zurich	891	17.8%	(17.2-18.5%)	24.0%	(21.0-27.3%)	0.95	(0.76-1.19)	0.87	(0.68-1.12)
East	698	13.0%	(12.4-13.6%)	23.1%	(19.4-27.2%)	0.91	(0.70-1.17)	0.90	(0.68-1.21)
Central	839	9.3%	(8.9-9.7%)	17.8%	(14.9-21.1%)	0.65	(0.51-0.84)	0.65	(0.49-0.86)
Ticino	605	4.9%	(4.6-5.1%)	32.0%	(27.9-36.3%)	1.42	(1.12-1.80)	1.44	(1.09-1.92)
**Residential area**				***p=0.0035***		***p=0.0036***		***p=0.2215***	
urban	4,734	74.4%	(73.2-75.6%)	26.7%	(25.2-28.3%)	1.00	(ref.)	1.00	(ref.)
rural	1,991	25.6%	(24.4-26.8%)	22.4%	(20.0-24.9%)	0.79	(0.67-0.93)	0.89	(0.74-1.07)
**Socioeconomic factors**									
**Education level**				***p=0.0905***		***p=0.0926***		***p=0.0041***	
compulsory	1,438	18.8%	(17.7-20.1%)	24.3%	(21.5-27.2%)	1.00	(ref.)	1.00	(ref.)
secondary	4,108	62.8%	(61.3-64.3%)	26.7%	(25.1-28.4%)	1.14	(0.95-1.36)	1.45	(1.16-1.81)
tertiary	1,179	18.3%	(17.2-19.6%)	23.3%	(20.5-26.3%)	0.95	(0.76-1.19)	1.44	(1.08-1.92)
**Income per capita**				***p=0.0000***		***p=0.0000***		***p=0.0271***	
lower tertile	2,058	32.4%	(30.9-33.9%)	19.8%	(17.7-22.0%)	1.00	(ref.)	1.00	(ref.)
middle tertile	2,121	35.2%	(33.7-36.8%)	26.5%	(24.3-28.8%)	1.46	(1.22-1.75)	1.10	(0.90-1.34)
upper tertile	2,073	32.3%	(30.9-33.8%)	29.8%	(27.5-32.3%)	1.72	(1.44-2.06)	1.32	(1.07-1.63)
unknown	473								
mean income (CHF per month)		4023	(3945-4101)						
**Hospitalisation insurance**				***p=0.0000***		***p=0.0000***		***p=0.0001***	
public	3,899	60.8%	(59.3-62.3%)	21.5%	(20.0-23.1%)	1.00	(ref.)	1.00	(ref.)
semiprivate	1,785	27.9%	(26.6-29.4%)	30.9%	(28.3-33.5%)	1.63	(1.40-1.90)	1.42	(1.19-1.70)
private	774	11.3%	(10.4-12.2%)	36.6%	(32.6-40.9%)	2.11	(1.72-2.58)	1.45	(1.15-1.83)
unknown	267								
**Healthcare-related factors and medication**									
**Osteoporosis medication**^**c**^**in the last 7 days**				***p=0.0000***		***p=0.0000***		***p=0.0000***	
none	5,316	82.5%	(81.3-83.6%)	20.5%	(19.3-21.9%)	1.00	(ref.)	1.00	(ref.)
self-ordered	24	0.3%	(0.2-0.4%)	43.7%	(23.5-66.3%)	3.00	(1.18-7.63)	1.83	(0.63-5.32)
prescribed (not including HRT)	448	6.2%	(5.6-7.0%)	79.9%	(74.9-84.1%)	15.37	(11.45-20.63)	11.14	(8.07-15.39)
prescribed HRT	740	11.1%	(10.1-12.1%)	38.0%	(33.7-42.6%)	2.38	(1.93-2.92)	1.62	(1.28-2.06)
unknown	197								
**Number of doctor visits in the past 12 months**				***p=0.0000***		***p=0.0000***		***p=0.0000***	
no visits	889	13.6%	(12.6-14.7%)	15.3%	(12.6-18.4%)	1.00	(ref.)	1.00	(ref.)
1 visit	1,339	22.2%	(20.9-23.5%)	18.7%	(16.4-21.3%)	1.28	(0.97-1.69)	1.49	(1.09-2.04)
2-6 visits	3,166	47.5%	(46.0-49.0%)	27.8%	(26.0-29.8%)	2.14	(1.67-2.73)	2.09	(1.58-2.76)
>6 visits	1,167	16.7%	(15.6-17.9%)	35.7%	(32.3-39.2%)	3.07	(2.34-4.03)	2.87	(2.10-3.92)
unknown	164								
mean number of visits		4.6	(4.3-4.8)						
**Gynaecologist check-up in the last 12 months**				***p=0.0043***		***p=0.0043***		***p=0.0000***	
no	3,618	51.8%	(50.3-53.3%)	23.8%	(22.1-25.5%)	1.00	(ref.)	1.00	(ref.)
yes	3,088	48.2%	(46.7-49.7%)	27.5%	(25.6-29.5%)	1.22	(1.06-1.39)	1.79	(1.51-2.12)
unknown	19								
**Cancer screening (breast, colon) ever performed**				***p=0.0000***		***p=0.0000***		***p=0.0000***	
no	2,978	47.5%	(46.0-49.0%)	16.7%	(15.1-18.4%)	1.00	(ref.)	1.00	(ref.)
yes	3,710	52.5%	(51.0-54.1%)	33.7%	(31.9-35.7%)	2.55	(2.20-2.95)	1.51	(1.27-1.79)
unknown	37								
**Health-oriented lifestyle**				***p=0.0242***		***p=0.0249***		***p=0.0050***	
no	379	7.2%	(6.4-8.2%)	20.3%	(15.7-25.7%)	1.00	(ref.)	1.00	(ref.)
yes	4,861	92.8%	(91.9-93.6%)	26.8%	(25.3-28.4%)	1.44	(1.05-1.98)	1.66	(1.16-2.35)
unknown	485								
**Self-assessed health state**				***p=0.0000***		***p=0.0000***		***p=0.0000***	
(very) good	5,414	81.8%	(80.6-82.9%)	23.0%	(21.6-24.3%)	1.00	(ref.)	1.00	(ref.)
medium	1,010	14.2%	(13.2-15.3%)	36.3%	(32.6-40.2%)	1.92	(1.60-2.30)	1.66	(1.33-2.08)
(very) poor	297	3.9%	(3.4-4.6%)	41.8%	(34.7-49.4%)	2.42	(1.77-3.30)	2.76	(1.88-4.06)
unknown	4								
**Risk factors**									
**Smoking (smoker)**				***p=0.0006***		***p=0.0006***		***p=0.5451***	
no	3,806	56.8%	(55.3-58.3%)	26.8%	(25.1-28.5%)	1.00	(ref.)	1.00	(ref.)
ex (former or not daily)	1,692	25.5%	(24.2-26.8%)	26.6%	(24.2-29.3%)	0.99	(0.85-1.16)	1.09	(0.91-1.31)
yes	1,225	17.7%	(16.6-19.0%)	20.2%	(17.7-23.1%)	0.69	(0.57-0.84)	0.97	(0.77-1.22)
unknown	2								
**Tobacco consumption per day**				***p=0.0000***		***p=0.0000***		***p=0.7711***	
none	5,328	79.9%	(78.6-81.1%)	27.1%	(25.6-28.6%)	1.00	(ref.)	1.00	(ref.)
0-9 cigarettes	541	8.0%	(7.2-8.9%)	18.8%	(15.2-23.0%)	0.62	(0.48-0.81)	0.91	(0.67-1.25)
≥10 cigarettes	836	12.1%	(11.2-13.2%)	20.8%	(17.6-24.3%)	0.71	(0.57-0.88)	0.93	(0.72-1.21)
unknown	20								
**Alcohol intake per day**				***p=0.8684***		***p=0.8670***		***p=0.6308***	
none	2,443	34.7%	(33.3-36.2%)	25.9%	(23.8-28.1%)	1.00	(ref.)	1.00	(ref.)
0-30 g	4,175	63.9%	(62.4-65.3%)	25.5%	(23.9-27.1%)	0.98	(0.85-1.13)	0.97	(0.82-1.14)
>30 g	92	1.4%	(1.1-1.8%)	23.2%	(14.6-34.9%)	0.87	(0.48-1.55)	0.74	(0.40-1.39)
unknown	15								
mean alcohol intake (g/d)		5.6	(5.3-5.8)						
**Underweight (BMI <20 kg/m**^**2**^**)**				***p=0.8712***		***p=0.8712***		***p=0.0146***	
no	5,694	86.3%	(85.2-87.4%)	25.7%	(24.4-27.1%)	1.00	(ref.)	1.00	(ref.)
yes	892	13.7%	(12.7-14.8%)	26.0%	(22.7-29.7%)	1.02	(0.83-1.24)	1.34	(1.06-1.69)
unknown	139								
mean BMI (kg/m^2^)		24.2	(24.0-24.3)						
**Physical activity**				***p=0.1262***		***p=0.1286***		***p=0.0398***	
inactive	1,596	20.9%	(19.8-22.2%)	27.5%	(24.9-30.4%)	1.00	(ref.)	1.00	(ref.)
partially active	2,675	41.5%	(40.0-43.0%)	24.2%	(22.2-26.2%)	0.84	(0.70-1.00)	1.06	(0.86-1.30)
active	2,442	37.6%	(36.1-39.1%)	26.2%	(24.1-28.3%)	0.93	(0.78-1.11)	1.27	(1.03-1.56)
unknown	12								
**Falls in the past 12 months**^**d**^				***p=0.0623***		***p=0.0625***		***p=0.0128***	
no	2,528	75.0%	(73.2-76.8%)	38.9%	(36.6-41.3%)	1.00	(ref.)	1.00	(ref.)
yes	892	25.0%	(23.2-26.8%)	43.3%	(39.4-47.4%)	1.20	(0.99-1.45)	1.30	(1.06-1.60)
unknown	3,305								

^a^ respondents, unweighted data.

^b^ controlled for sociodemographic and socioeconomic factors (age, region and residential area, income, hospitalisation insurance type, and education level) except language region was not controlled for region because of collinearity.

^c^ information only available for women older than 40 years.

^d^ information only available for women aged 60 years and older.

BDM ever performed in women aged 40 years and older living in Switzerland according to sociodemographic, socioeconomic, risk and healthcare- related factors (SHS 2007 data).

#### Factors associated with BDM utilisation

Most of the sociodemographic factors (age, language region, region, and nationality), all the socioeconomic factors (education level, income, and hospitalisation insurance type) and all the healthcare-related factors (number of doctor visits, gynaecologist check-up, cancer screening ever performed, health-oriented lifestyle, and self-assessed health) including OM showed statistically significant associations with BDM utilisation after adjusting for sociodemographic and socioeconomic factors, but only few of the assessed osteoporosis risk factors (underweight, physical activity level, and history of falls) did (Table 1).

Lifetime BDM prevalence increased with age but decreased slightly in those 80 years and older compared to those aged 70–79 years. Women from the French-speaking (OR 1.58) and the Italian-speaking (OR 1.56) region more often had ever had a BDM than those from the German-speaking region. Furthermore, Swiss dual citizens more often had a BDM than did Swiss women (OR 1.43). This association was only evident when adjusting for sociodemographic factors. On the other hand, after adjustment for sociodemographic and socioeconomic factors, residential area was no longer associated with BDM.

The associations of BDM prevalence with income and hospitalisation insurance type remained statistically significant even though the strength of the associations were reduced when controlling for sociodemographic and socioeconomic factors. In contrast to the crude results in which no association was observed between education level and BDM utilisation, the adjusted results showed that women with a secondary (OR 1.45) or tertiary (OR 1.44) education more often had a BDM than women with only compulsory education. Furthermore, women with a health-oriented lifestyle (OR 1.66) and those who had already undergone a screen for breast or colon cancer (OR 1.51) had a higher BDM prevalence. Similar associations were found for gynaecologist check-up (OR 1.79) and number of doctor visits.

With regard to osteoporosis risk factors, lifetime BDM prevalence was no longer associated with smoking after adjustment for sociodemographic factors. On the other hand, only in the adjusted analyses, underweight women (OR 1.34) and women with a history of falls (OR 1.30) – and thus those with elevated osteoporosis and osteoporotic fracture risk – had more often undergone a BDM. Women with a higher physical activity level and thus a decreased osteoporosis risk however were also more likely to have had a BDM than were inactive women (OR 1.27).

### Osteoporosis medication

Of the 7,052 women older than 40 years, 216 were excluded due to missing answers regarding OM in the last 7 days, leaving 6,836 women for analysis (96.9%).

#### Prevalence of current OM medication

In 2007, 7.8% (95% CI: 7.0-8.6%) of female Swiss residents older than 40 years reported having taken prescribed OM (not including HRT) in the 7 days prior to the interview (Table 2). OM prevalence increased with age and was highest in the 70–79 years age group (17.7%) and lowest in the 40–49 years age group (1.5%). The prevalence was highest in the French-speaking region of Switzerland (9.3%). OM prevalence was higher in less educated women and in women with private hospitalisation insurance (10.3%). Similarly, higher proportions were observed in women with more doctor visits in the past year and in women who had ever undergone a breast or colon cancer screen (9.0%).

**Table 2:** OM in women according to sociodemographic, socioeconomic, and healthcare-related factors

**Osteoporosis medication**	**Sample**^**a**^	**Population**		** Proportion with OM**		** Unadjusted model**		** Adjusted model**^**b**^	
**HRT not included**	**N**	**%**	**(95% CI)**	**%**	**(95% CI)**	**crude OR**	**(95% CI)**	**adjusted OR**	**(95% CI)**
**Total**	6,836	100.0%		7.8%	(7.0-8.6%)				
**Sociodemographic factors**									
**Age group**				***p=0.0000***		***p=0.0000***		***p=0.0000***	
40-49 years	1,645	29.3%	(27.8-30.8%)	1.5%	(0.9-2.5%)	0.27	(0.15-0.51)	0.22	(0.11-0.45)
50-59 years	1,586	25.3%	(23.9-26.7%)	5.1%	(3.9-6.8%)	1.00	(ref.)	1.00	(ref.)
60-69 years	1,667	21.5%	(20.3-22.7%)	9.2%	(7.7-11.0%)	1.87	(1.31-2.67)	1.93	(1.32-2.83)
70-79 years	1,258	15.4%	(14.4-16.4%)	17.7%	(15.1-20.6%)	3.96	(2.79-5.61)	4.07	(2.75-6.01)
≥80 years	680	8.6%	(7.9-9.4%)	15.5%	(12.4-19.2%)	3.38	(2.28-5.01)	3.53	(2.27-5.47)
mean age (years)		59.3	(58.9-59.7)						
**Nationality**				***p=0.7348***		***p=0.6753***		***p=0.6460***	
Swiss citizen	5,565	76.7%	(75.3-78.1%)	7.9%	(7.1-8.8%)	1.00	(ref.)	1.00	(ref.)
Swiss dual citizen	721	10.0%	(9.2-10.9%)	6.8%	(5.0-9.4%)	0.85	(0.59-1.23)	1.09	(0.74-1.61)
others	548	13.3%	(12.0-14.6%)	7.5%	(5.0-11.0%)	0.94	(0.61-1.45)	1.28	(0.73-2.25)
unknown	2								
**Language region**				***p=0.0452***		***p=0.0774***		***p=0.1380***	
German	4,179	72.3%	(71.5-73.0%)	7.3%	(6.4-8.3%)	1.00	(ref.)	1.00	(ref.)
French	2,034	22.6%	(21.9-23.3%)	9.3%	(7.9-11.0%)	1.31	(1.03-1.65)	1.22	(0.94-1.59)
Italian	623	5.1%	(4.8-5.4%)	7.6%	(5.5-10.4%)	1.05	(0.72-1.52)	0.84	(0.57-1.25)
**Region**				***p=0.0041***		***p=0.0067***		***p=0.0259***	
Lake Geneva	1,235	17.5%	(16.9-18.2%)	8.9%	(7.2-10.9%)	0.93	(0.68-1.28)	0.91	(0.64-1.30)
Midland	1,791	23.2%	(22.5-24.0%)	9.5%	(7.8-11.6%)	1.00	(ref.)	1.00	(ref.)
Northwest	762	14.8%	(14.3-15.4%)	9.0%	(7.0-11.6%)	0.95	(0.66-1.35)	0.97	(0.66-1.41)
Zurich	891	17.6%	(16.9-18.3%)	5.9%	(4.4-7.7%)	0.59	(0.41-0.86)	0.61	(0.41-0.92)
East	699	12.7%	(12.1-13.2%)	6.0%	(4.3-8.3%)	0.61	(0.40-0.92)	0.69	(0.44-1.06)
Central	842	9.3%	(8.9-9.7%)	5.2%	(3.6-7.5%)	0.52	(0.33-0.81)	0.52	(0.32-0.83)
Ticino	616	4.9%	(4.7-5.2%)	7.9%	(5.8-10.8%)	0.82	(0.55-1.24)	0.68	(0.44-1.06)
**Residential area**				***p=0.2792***		***p=0.2796***		***p=0.3755***	
urban	4,814	74.7%	(73.5-75.9%)	8.0%	(7.2-9.0%)	1.00	(ref.)	1.00	(ref.)
rural	2,022	25.3%	(24.1-26.5%)	7.0%	(5.6-8.7%)	0.86	(0.66-1.13)	0.87	(0.65-1.18)
**Socioeconomic factors**									
**Education level**				***p=0.0038***		***p=0.0044***		***p=0.5559***	
compulsory	1,562	21.1%	(19.9-22.4%)	10.0%	(8.2-12.2%)	1.00	(ref.)	1.00	(ref.)
secondary	4,109	61.3%	(59.8-62.8%)	7.5%	(6.5-8.5%)	0.72	(0.56-0.93)	0.85	(0.62-1.17)
tertiary	1,161	17.6%	(16.5-18.8%)	5.9%	(4.6-7.7%)	0.57	(0.40-0.81)	0.94	(0.62-1.45)
unknown	4								
**Income per capita**				***p=0.9508***		***p=0.9579***		***p=0.8105***	
lower tertile	2,075	32.8%	(31.4-34.4%)	7.8%	(6.4-9.5%)	1.00	(ref.)	1.00	(ref.)
middle tertile	2,148	35.4%	(33.9-37.0%)	7.5%	(6.4-8.9%)	0.96	(0.72-1.28)	0.90	(0.67-1.23)
upper tertile	2,090	31.8%	(30.3-33.2%)	7.5%	(6.3-9.0%)	0.96	(0.72-1.29)	0.94	(0.67-1.32)
unknown	523								
mean income (CHF per month)		3995	(3918-4072)						
**Hospitalisation insurance**				***p=0.0020***		***p=0.0026***		***p=0.0270***	
public	3,961	61.5%	(60.0-63.0%)	6.7%	(5.8-7.7%)	1.00	(ref.)	1.00	(ref.)
semiprivate	1,795	27.2%	(25.9-28.6%)	9.1%	(7.7-10.8%)	1.40	(1.10-1.79)	1.40	(1.07-1.84)
private	794	11.3%	(10.4-12.3%)	10.3%	(8.0-13.2%)	1.61	(1.17-2.22)	1.40	(0.98-1.98)
unknown	286								
**Healthcare-related factors and medication**									
**Bone density measurement**				***p=0.0000***		***p=0.0000***		***p=0.0000***	
no	4,670	73.8%	(72.5-75.1%)	2.2%	(1.8-2.8%)	1.00	(ref.)	1.00	(ref.)
yes	1,858	26.2%	(24.9-27.6%)	22.8%	(20.6-25.3%)	13.03	(10.03-16.92)	9.87	(7.37-13.22)
unknown	308								
**Number of doctor visits in the past 12 months**				***p=0.0000***		***p=0.0000***		***p=0.0000***	
no visits	896	13.5%	(12.5-14.7%)	2.9%	(1.8-4.7%)	1.00	(ref.)	1.00	(ref.)
1 visit	1,333	21.6%	(20.3-22.9%)	3.8%	(2.8-5.2%)	1.31	(0.74-2.33)	1.78	(0.97-3.27)
2-6 visits	3,206	47.4%	(45.8-48.9%)	8.4%	(7.2-9.7%)	3.02	(1.83-5.01)	3.01	(1.75-5.16)
>6 visits	1,227	17.5%	(16.4-18.7%)	13.8%	(11.6-16.4%)	5.32	(3.16-8.94)	4.79	(2.74-8.39)
unknown	174								
mean number of visits		4.7	(4.4-5.0)						
**Gynaecologist check-up in the last 12 months**				***p=0.3969***		***p=0.3971***		***p=0.0001***	
no	3,621	52.5%	(50.9-54.0%)	7.8%	(6.8-9.0%)	1.00	(ref.)	1.00	(ref.)
yes	3,017	47.5%	(46.0-49.1%)	7.2%	(6.2-8.3%)	0.91	(0.73-1.13)	1.61	(1.26-2.06)
unknown	198								
**Cancer screening (breast, colon) ever performed**				***p=0.0002***		***p=0.0002***		***p=0.4590***	
no	2,854	46.2%	(44.7-47.8%)	6.0%	(5.0-7.1%)	1.00	(ref.)	1.00	(ref.)
yes	3,740	53.8%	(52.2-55.3%)	9.0%	(8.0-10.1%)	1.56	(1.24-1.96)	1.10	(0.85-1.42)
unknown	242								
**Health-oriented lifestyle**				***p=0.3006***		***p=0.3019***		***p=0.2900***	
no	374	7.2%	(6.3-8.1%)	5.9%	(3.7-9.2%)	1.00	(ref.)	1.00	(ref.)
yes	4,796	92.8%	(91.9-93.7%)	7.5%	(6.7-8.4%)	1.30	(0.79-2.15)	1.36	(0.77-2.42)
unknown	1,666								
**Self-assessed health state**				***p=0.0000***		***p=0.0000***		***p=0.0000***	
(very) good	5,406	79.9%	(78.6-81.1%)	5.9%	(5.2-6.6%)	1.00	(ref.)	1.00	(ref.)
medium	1,080	15.2%	(14.2-16.3%)	14.6%	(12.1-17.4%)	2.74	(2.13-3.53)	2.02	(1.52-2.68)
(very) poor	347	4.9%	(4.3-5.6%)	17.4%	(12.3-24.2%)	3.40	(2.20-5.24)	2.89	(1.64-5.09)
unknown	3								

^a^ respondents, unweighted data.

^b^ controlled for sociodemographic and socioeconomic factors (age, region, residential area, income, hospitalisation insurance type, and education level) except language region was not controlled for region because of collinearity.

Taking prescribed OM in the last 7 days in women older than 40 years living in Switzerland according to sociodemographic, socioeconomic and healthcare-related factors (SHS 2007 data).

#### Factors associated with prescribed OM utilisation

Most of the healthcare-related factors (number of doctor visits, gynaecologist check-up, BDM ever performed, and self-assessed health) showed a statistically significant association with OM when controlling for sociodemographic and socioeconomic factors, but only few of the sociodemographic factors (age, and region) and only one socioeconomic factor (hospitalisation insurance type) did (Table 2): Prescribed OM prevalence was much higher in women having ever had a BDM (OR 9.87), although OM can be taken even without having performed a BDM, and prevalence increased with number of physician visits in the past year. In addition, prescribed OM utilisation was higher in those having had a gynaecologist check- up in the last 12 months (OR 1.61) after adjustment for sociodemographic factors, while no association was observed in the crude results.

On the other hand and in contrast to the crude association, education level and breast or colon cancer screening were no longer associated with medication when adjusting for sociodemographic factors.

## Discussion

According to our analysis of the SHS 2007 data, lifetime prevalence of BDM and current OM in women aged at least 40 years was only 26% and 8%, respectively. These prevalences seem low taking into account that BDM is not only performed to screen but also to diagnose osteoporosis after a fracture occurred, and similarly that OM is not only taken to cure but also to prevent osteoporosis. This low coverage might indicate that the risk for osteoporosis is not fully appreciated by physicians or the general population in Switzerland. The public seems to perceive osteoporosis as a minor health issue, for example compared to cancer, although osteoporosis is estimated to account for more disability-adjusted life-years lost than most cancers, except lung cancer [26].

In Switzerland, healthcare services are in principle equally accessible to all individuals, as the healthcare system is publicly funded, and health insurance, being compulsory, covers all residents equally. Nevertheless, we found disparities in the utilisation of BDM and OM with respect to sociodemographic, socioeconomic and healthcare-related factors (Table 1, Table 2). Similar variations have previously been documented for other curative and preventive healthcare services in Switzerland [20,27]. Inequalities in BDM utilisation have also been observed in the US and in Canada, despite existing recommendations for routine BDM [9,11,12].

### Sociodemographic, socioeconomic and healthcare-related factors

Generally, the utilisation of preventive health care services is lower among foreigners than among Swiss nationals [20], although no differences were found for mammography screening, regarding nationality [27,28]. Similarly we found that BDM prevalence was comparable for foreign and for Swiss women, but unexpectedly was higher for Swiss dual citizens.

Consistent with general health service demand [20] and particularly with mammography screening [27,29,30] in Switzerland, also BDM and OM utilisation in women differed by region and/or language region. These disparities may not only be caused by unequal needs or physician recommendations, but also by differences in physician density or, with respect to BDM, due to differences in the density and/or distribution of DXA centres [31], and therefore unequal access.

Also similar to the pattern found for the general use of health services [20] and mammography screening [27] our results showed that women with supplementary private insurance in case of hospitalisation utilised BDM and OM more often. This cannot be explained by the fact that having private insurance is also correlated with higher income and higher education level because we controlled for both factors. But several other reasons may account for this remarkable variation by type of insurance. On the one hand, women′s demand e.g. due to more worrying, their needs or the consent for BDM or medication could differ. On the other hand, doctors’ provision of care, especially of BDM, may also have differed because compulsory health insurance does not cover BDM for screening purposes, except for those with certain major risk factors, whereas all the BDM for diagnostic purposes are reimbursed [10]. BDM screening is to some extent covered for individuals who have an optional health insurance supplement. In contrast to BDM, reimbursement for OM is not dependent on health insurance type but on having had a BDM: With the exception of calcium, vitamin D, and some bisphosphonates, all osteoporosis drugs are only reimbursed if the person has BDM-diagnosed osteoporosis or has had a fracture [19].

Furthermore, consistent with the general demand [20] but partially in contrast to mammography utilisation [27,28] we found that BDM prevalence increased with income and education level in women. Similarly, in Canada higher income was directly associated with higher BDM utilisation in women [14], whereas in the US a similar disparity existed for screening but not for diagnostic BDM [15]. It is unlikely that differences in osteoporosis prevalence explain the observed variations by socioeconomic status [32].

Similar to the findings for mammography screening in Switzerland [27], our results clearly show that history of BDM and taking OM increased by the intensity of healthcare use in women, as quantified by the number of doctor visits in the 12 months prior to the interview or having had a gynaecologist check-up in the last 12 months. Likewise in the US, postmenopausal women with diagnosed osteoporosis were more likely to receive osteoporosis treatment when they had routine medical care or when they had discussed the treatment with a gynaecologist [18]. A higher healthcare use could obviously be the consequence of a poorer health state, or alternatively could result from a health-oriented but worrying health behaviour independent from health state. However increased anxiety in the absence of osteoporosis risk factors does not seem to be associated with a reduced bone density, and therefore would not constitute an indication for performing a BDM [33]. Our findings might indicate an inappropriate BDM and OM utilisation in that certain women have BDM or OM who do not meet the indications, and others who are likely to meet the indications do not get it. BDM and OM should be targeted according to established recommendations [34] to make rational use of the healthcare budget and to properly balance benefits and harms of BDM and OM [35].

The variation of BDM and OM utilisation by sociodemographic, socioeconomic and healthcare-related factors, which possibly originate from variations in reimbursement and in availability and utilisation of health services could lead to a situation in which some women are underserved or overtreated. This might foster further health inequalities and therefore needs further investigation.

### Osteoporosis risk factors

According to the SVGO recommendations [9], BDM is indicated for women and men with at least one major or two minor risk factors for osteoporosis or an osteoporotic fracture. The major factors include being older than 70 years, being underweight and having pronounced physical inactivity. The minor factors include smoking and excessive alcohol consumption.

In Swiss resident women, all major but none of the minor risk factors were associated with ever having had a BDM. This may reflect the designated consideration of the SVGO recommendations to decide who should have a BDM, or it may also result from specific conditions when BDM is covered by the health insurance [10].

Osteoporosis prevalence and estimated 10-year osteoporosis facture probability increase with age, i.e. probability for women in Switzerland is 6% at age 50 but 33% at age 85 [1]. Hence, an age-dependent increase of BDM and OM utilisation were expected, but we found a slight decrease for individuals aged 80 years and older compared to those aged 70–79 years. This is inconsistent with the SVGO recommendations as age is a major risk factor [36]. However, this old-age effect may result from selection bias, as institutionalised women are not covered by the SHS. Osteoporotic fractures are a major factor leading to people having to enter a nursing home. [2,37-39]. It is therefore likely that women with BDM-diagnosed osteoporosis and subsequent OM are more likely residing in such institutions, which is especially true for the oldest.

### Strengths and limitations

This study was based on the SHS data, which makes the results representative for adult Swiss resident women. The SHS data provide rich information on health-related issues and on the sociodemographic and socioeconomic characteristics of the respondents.

Although the participants were selected in a random two-stage sampling process, selection bias cannot be excluded, as only persons living in private households with a landline phone could be surveyed. I.e., as institutionalised Swiss residents were not covered by the SHS, this study may have underestimated the population prevalence of lifetime BDM and current OM [2,37]. Additionally, some misclassification due to reporting and recall bias may have occurred as the data were based on information self-reported by the participants and therefore were dependent on their understanding of the questionnaire, their recall capacity and their willingness to respond accurately.

Either, BDM is used for osteoporosis screening, for diagnosis after osteoporotic fractures, or to monitor medical osteoporosis treatment. The SHS 2007 asked about BDM history but not about motive for the most recently performed BDM. Therefore, no differentiation between BDM for screening, diagnosis, or monitoring was possible. Similarly, for OM, it was not possible to distinguish treatment for primary prevention of an osteoporotic fracture and therapy for secondary prevention of further fractures. Finally, the questionnaire included no questions on osteoporosis or osteoporotic fractures.

## Conclusions

Our analysis of the SHS 2007 data describes for the first time factors associated with BDM history and current OM utilisation in Swiss women. The low BDM and OM coverage might indicate that the risk of osteoporosis and osteoporotic fractures is not fully appreciated by physicians or the general population in Switzerland. BDM and OM prevalence varied substantially according to healthcare-related, sociodemographic, and socioeconomic factors, which might foster further health inequalities. The reasons for these disparities and the low BDM and OM prevalences should be investigated in further studies of elderly Swiss women, including those living in institutions.

## Abbreviations

BDM: Bone mineral density measurement; CATI: Computer-assisted phone interview; DXA: Dual-energy x-ray absorptiometry; HRT: Hormone replacement therapy; OM: Osteoporosis medication; SFSO: Swiss Federal Statistical Office; SHS: Swiss Health Survey; SVGO: Swiss Union against Osteoporosis.

## Competing interests

This work of Rita Born was performed to fulfil the requirements to obtain a Master of Public Health degree. Marcel Zwahlen was the supervisor of this master thesis. Marcel Zwahlen^′^s position was funded from 2009-2012 by the Swiss School of Public Health (http://www.ssphplus.ch). Otherwise, no specific funding was available for this work.

## Authors' contributions

RB conceived the design of the study, performed the statistical analysis and drafted the manuscript. MZ participated in the design of the study, advised on how to perform the statistical analysis and revised the manuscript. Both authors read and approved the final manuscript.

## Pre-publication history

The pre-publication history for this paper can be accessed here:

http://www.biomedcentral.com/1471-2474/14/10/prepub

## References

[B1] Lippuner K Johansson H Kanis JA Rizzoli R Remaining lifetime and absolute 10-year probabilities of osteoporotic fracture in Swiss men and women Osteoporos Int 2009 20 7 1131 1140 10.1007/s00198-008-0779-8 18974918

[B2] Federal Office of Public Health Osteoporose und Stürze im Alter 2004 1 84

[B3] Schwenkglenks M Lippuner K Häuselmann HJ Szucs TD A model of osteoporosis impact in Switzerland 2000–2020 Osteoporos Int 2005 16 6 659 671 10.1007/s00198-004-1743-x 15517190

[B4] Kanis JA Burlet N Cooper C Delmas P European guidance for the diagnosis and management of osteoporosis in postmenopausal women Osteoporos Int 2008 19 4 399 428 10.1007/s00198-008-0560-z 18266020 PMC2613968

[B5] Siris ES Baim S Nattiv A Primary care use of FRAX: absolute fracture risk assessment in postmenopausal women and older men Postgrad Med 2010 122 1 82 90 10.3810/pgm.2010.01.2102 20107292

[B6] World Health Organisation FRAX Fracture Risk Assessment: Calculation Tool for Switzerland http://www.shef.ac.uk/FRAX/tool.jsp?country=15

[B7] Schwenkglenks M Lippuner K Simulation-based cost-utility analysis of population screening-based alendronate use in Switzerland Osteoporos Int 2007 18 11 1481 1491 10.1007/s00198-007-0390-4 17530156

[B8] Lippuner K Popp A Szucs T Schwenkglenks M Kosteneffektivität einer Osteoporose- Behandlung nach systematischem Bevölkerungsscreening in der Schweiz: Auswirkungen der Preisreduktion des Alendronat-Originalpräparates Pharmaco Economics 2008 6 1 19 28

[B9] Swiss Union Against Osteoporosis Empfehlung zur Osteoporose Diagnostik, Prävention, Behandlung 2003 1 23

[B10] Federal Department of Home Affairs SR 832.112.31 Krankenpflege- Leistungsverordnung KLV Art.1: Pflichtleistungen der Krankenkassen, Anhang 1 2001

[B11] Brown JP Josse RG Scientific Advisory Council of the Osteoporosis Society of Canada 2002 Clinical practice guidelines for the diagnosis and management of osteoporosis in Canada Can Med Assoc J 2002 167 10 Suppl S1 S34 12427685 PMC134653

[B12] U. S. Preventive Services Task Force Screening for osteoporosis in postmenopausal women: recommendations and rationale Ann Intern Med 2002 137 6 526 528 12230355 10.7326/0003-4819-137-6-200209170-00014

[B13] U. S. Preventive Services Task Force Screening for osteoporosis: U.S. Preventive services task force recommendation statement Ann Intern Med 2011 154 5 356 364 21242341 10.7326/0003-4819-154-5-201103010-00307

[B14] Demeter S Leslie WD Lix L MacWilliam L Finlayson GS Reed M The effect of socioeconomic status on bone density testing in a public health-care system Osteoporos Int 2007 18 2 153 158 10.1007/s00198-006-0212-0 17019518

[B15] Neuner JM Zhang X Sparapani R Laud PW Nattinger AB Racial and socioeconomic disparities in bone density testing before and after hip fracture J Gen Intern Med 2007 22 9 1239 1245 10.1007/s11606-007-0217-1 17594131 PMC2219762

[B16] Nayak S Roberts MS Greenspan SL Factors associated with diagnosis and treatment of osteoporosis in older adults Osteoporos Int 2009 20 11 1963 1967 10.1007/s00198-008-0831-8 19151910 PMC2765627

[B17] Vanasse A Dagenais P Niyonsenga T Grégoire JP Courteau J Hemiari A Bone mineral density measurement and osteoporosis treatment after a fragility fracture in older adults: regional variation and determinants of use in Quebec BMC Musculoskelet Disord 2005 6 33 10.1186/1471-2474-6-33 15969760 PMC1187894

[B18] Brennan RM Wactawski-Wende J Crespo CJ Dmochowski J Factors associated with treatment initiation after osteoporosis screening Am J Epidemiol 2004 160 5 475 483 10.1093/aje/kwh245 15321845

[B19] Federal Office of Public Health List of pharmaceutical specialities LS 2011

[B20] Bisig B Gutzwiller F Gesundheitswesen Schweiz: gibt es Unter- oder Überversorgung? Die Bedeutung von Sozialschicht, Wohnregion, Nationalität, Geschlecht und Versicherungstatus NFP45 Rüegger Verlag 2004 1 1 247

[B21] Swiss Federal Statistical Office Rapport de méthodes - Enquête suisse sur la santé 2007 2010 1 68

[B22] Swiss Federal Statistical Office Schweizerische Gesundheitsbefragung 2007 in Kürze: Konzept, Methode, Durchführung 2008 1 9

[B23] Swiss Federal Statistical Office Schweizerische Gesundheitsbefragung 2007: Telefonischer und schriftlicher Fragebogen 2010 1 126

[B24] Swiss Federal Statistical Office Schweizerische Gesundheitsbefragung 2007: Die Indizes 2010 1 89

[B25] Federal Council SR 431.012.1 Verordnung vom 30. Juni 1993 über die Durchführung von statistischen Erhebungen des Bundes (Statistikerhebungsverordnung) 1993

[B26] Johnell O Kanis JA An estimate of the worldwide prevalence and disability associated with osteoporotic fractures Osteoporos Int 2006 17 12 1726 1733 10.1007/s00198-006-0172-4 16983459

[B27] Schmidlin K Wie häufig und wie unterschiedlich werden Krebsvorsorgeuntersuchungen von der Schweizerischen Bevölkerung verwendet: Eine Auswertung der Schweizerischen Gesundheitsbefragung CDSP Saphir 2002 2006 1 57

[B28] Fontana M Bischoff A Uptake of breast cancer screening measures among immigrant and Swiss women in Switzerland Swiss Med Wkly 2008 138 49–50 752 758 19130329 10.4414/smw.2008.12328

[B29] Wanner P Raymond L Bouchardy C Geographical disparities in self-reported use of mammography and breast self-examination according to the Swiss health survey Ann Oncol 2001 12 4 573 574 10.1023/A:1011147027410 11398895

[B30] Keller B Stutz EZ Tibblin M Ackermann-Liebrich U Faisst K Probst-Hensch N Screening mammographies in Switzerland: what makes female and male physicians prescribe them? Swiss Med Wkly 2001 131 21–2 311 319 11584693 10.4414/smw.2001.09710

[B31] Register of DEXA centres in Switzerlandhttp://www.dexa.ch/

[B32] Wang MC Dixon LB Socioeconomic influences on bone health in postmenopausal women: findings from NHANES III, 1988–1994 Osteoporos Int 2006 17 1 91 98 10.1007/s00198-005-1917-1 15883659

[B33] Mulherin DM Smith JA Price T Is patient anxiety about osteoporosis sufficient indication for measuring bone mineral density? Clin Exp Rheumatol 2000 18 6 789 11138355

[B34] Swiss Union Against Osteoporosis Empfehlung zur Osteoporose Prävention Diagnostik Behandlung 2010 1 16

[B35] Allemann S Saner C Zwahlen M Christ ER Diem P Stettler C Long-term cardiovascular and non-cardiovascular mortality in women and men with type 1 and type 2 diabetes mellitus: a 30-year follow-up in Switzerland Swiss Med Wkly 2009 139 39–40 576 583 19838876 10.4414/smw.2009.12785

[B36] Nayak S Roberts MS Greenspan SL Factors associated with osteoporosis screening and recommendations for osteoporosis screening in older adults J Gen Intern Med 2009 24 5 585 591 10.1007/s11606-009-0936-6 19274478 PMC2669865

[B37] Holroyd C Cooper C Dennison E Epidemiology of osteoporosis Best Pract Res Clin Endocrinol Metab 2008 22 5 671 685 10.1016/j.beem.2008.06.001 19028351

[B38] Suhm N Lamy O Lippuner K OsteoCare study group Management of fragility fractures in Switzerland: results of a nationwide survey Swiss Med Wkly 2008 138 45–6 674 683 18855150 10.4414/smw.2008.12294

[B39] Schürch MA Rizzoli R Mermillod B Vasey H Michel JP Bonjour JP A prospective study on socioeconomic aspects of fracture of the proximal femur J Bone Miner Res 1996 11 12 1935 1942 8970896 10.1002/jbmr.5650111215

